# Efficacy of Limosilactobacillus reuteri UBLRu-87 in Infantile Colic and Its Symptoms: A Double-Blind, Placebo-Controlled Study

**DOI:** 10.7759/cureus.81856

**Published:** 2025-04-07

**Authors:** Rajesh Venkataraman, Mahendrappa Kotyal Basvanyappa, Brajogopal Samanta, Mahalaxmi Yadla, Naga Amrutha Ravi, Jayanthi Neelamraju, Ratna Sudha Madempudi

**Affiliations:** 1 Pharmacology, Adichunchanagiri University, Karnataka, IND; 2 Pediatrics, Adichunchanagiri Hospital and Research Center, Adichunchanagiri University, Karnataka, IND; 3 Department of Life Sciences, GITAM School of Science, Gandhi Institute of Technology and Management (GITAM), Visakhapatnam, IND; 4 Centre for Research and Development, Unique Biotech Ltd., Hyderabad, IND

**Keywords:** colic pain, gut microbiota, infants, lactobacillus reuteri, probiotics

## Abstract

Infantile colic, characterized by excessive crying in healthy infants, presents a significant challenge for parents and healthcare providers. This study investigated the potential of *Limosilactobacillus reuteri *(*L. reuteri*)UBLRu-87 in alleviating colic symptoms and enhancing parental quality of life (QOL) in eighty breastfed infants ≤4 months of age through a double-blind, placebo-controlled trial. The treatment group received *L. reuteri* UBL Ru-87 drops, whereas the placebo group received an identical-looking placebo. Over 28 days, the study assessed crying time, burping, facial flushing, and fussiness. Parental QOL was evaluated using a visual analog scale (VAS). Fecal samples were analyzed for microbial diversity before and after treatment. Results demonstrated a significant reduction in colic symptoms in the probiotic group, particularly in crying time. In addition, burping, fussiness, and facial flushing were reduced significantly in probiotic-treated infants. Responders (infants with a 50% reduction in crying time) were also significantly enhanced. Parents of infants in the probiotic group reported a considerable improvement in QOL. Fecal microbiome analysis indicated beneficial shifts in microbial composition in the probiotic group, including increased Bifidobacteria and Firmicutes, along with reduced harmful bacteria like *Enterococci*, *Staphylococci*, and *Streptococci*. In conclusion, the use of *L. reuteri* UBLRu-87 notably diminished colic symptoms and improved parental QOL. These results suggest that *L. reuteri* UBLR-87 holds promise as a therapeutic option for alleviating infantile colic pain by influencing gut microbiota.

## Introduction

Colic pain in infants often manifests as repeated inconsolable crying episodes that persist for a prolonged period. The modified Wessel’s criteria define infantile colic as crying and/or fussing for more than three hours a day, occurring for more than three days in a week, without any apparent reason, and being unpreventable or unstoppable by the caregiver [1]. Several studies have shown that probiotic intake can significantly reduce the crying time of newborn babies [2-4]. Probiotics help modulate the gut microbiota by restoring a healthy microbiome. It is suggested that colicky infants have gut microbiota dysbiosis with less diversity, barrier alterations, and mild chronic gastrointestinal inflammation as compared to healthy infants with no colic [5,6]. 

A few reports have indicated that infants experiencing colic pain show an increased presence of Proteobacteria, including* Escherichia, Klebsiella, *and* Pseudomonas, *and a decreased presence of *Bifidobacteria, *whereas those without colic tend to have higher levels of Firmicutes and Actinobacteria phyla in their fecal composition [7]. Most studies on probiotics in infants with colic pain have focused on *Limosilactobacillus reuteri *(earlier *Lactobacillus reuteri* (*L. reuteri*) and have proven its effectiveness in reducing the crying time in colicky babies [8-11]. However, some studies with *L. reuteri *have also reported it to be ineffective in colic management [12]. As the effects of probiotics are strain-specific, it was of interest to investigate the efficacy of *L. reuteri *UBLRu-87 on colic pain in infants fed with breastmilk. The probiotic strain *L. reuteri *UBLRu-87 is a well-characterized and safe strain [13] found to be efficacious in the treatment of periodontitis. Moreover, it has been included as part of multi-strain probiotic formulations, which helped alleviate irritable bowel syndrome and bacterial vaginosis in clinical trials [14,15]. In an *in vitro* study on survivability in the gastrointestinal tract, *L. reuteri* UBLRu-87 showed 100% viability for two hours in the gut model of the stomach. At 24 hours of colonic incubation, viability further increased [16]. In the present study, the efficacy of* L. reuteri *UBLRu-87 in breastfed infants with colic pain is reported.

## Materials and methods

Study design

This double-blind, randomized, placebo-controlled study was conducted from September 2021 to July 2022 at Adichunchanagiri Hospital & Research Center (AH & RC), Adichunchanagiri University, India. The study was approved by the Institutional Ethics Committee of AH & RC, Mandya Bengaluru, Karnataka (IEC/AH &RC/AC/020/2021). The study adhered to the code of conduct for research involving human volunteers, as specified by the International Conference on Harmonisation-Good Clinical Practice (ICH-GCP), the Indian Council of Medical Research guidelines (ethical guidelines for biomedical research on human subjects), and the principles of the Declaration of Helsinki. Informed consent forms were duly approved by the ethical committee of the hospital, and the trial was prospectively registered with the Clinical Trials Registry, India (CTRI Reg. No: CTRI/2021/09/036378). The study was initiated only after obtaining informed consent from the caregivers/ parent or a legal guardian by the study team. Parents or caregivers of participating infants were free to withdraw their infant from the study or decline participation at any time.

Study Population

**Figure 1:**
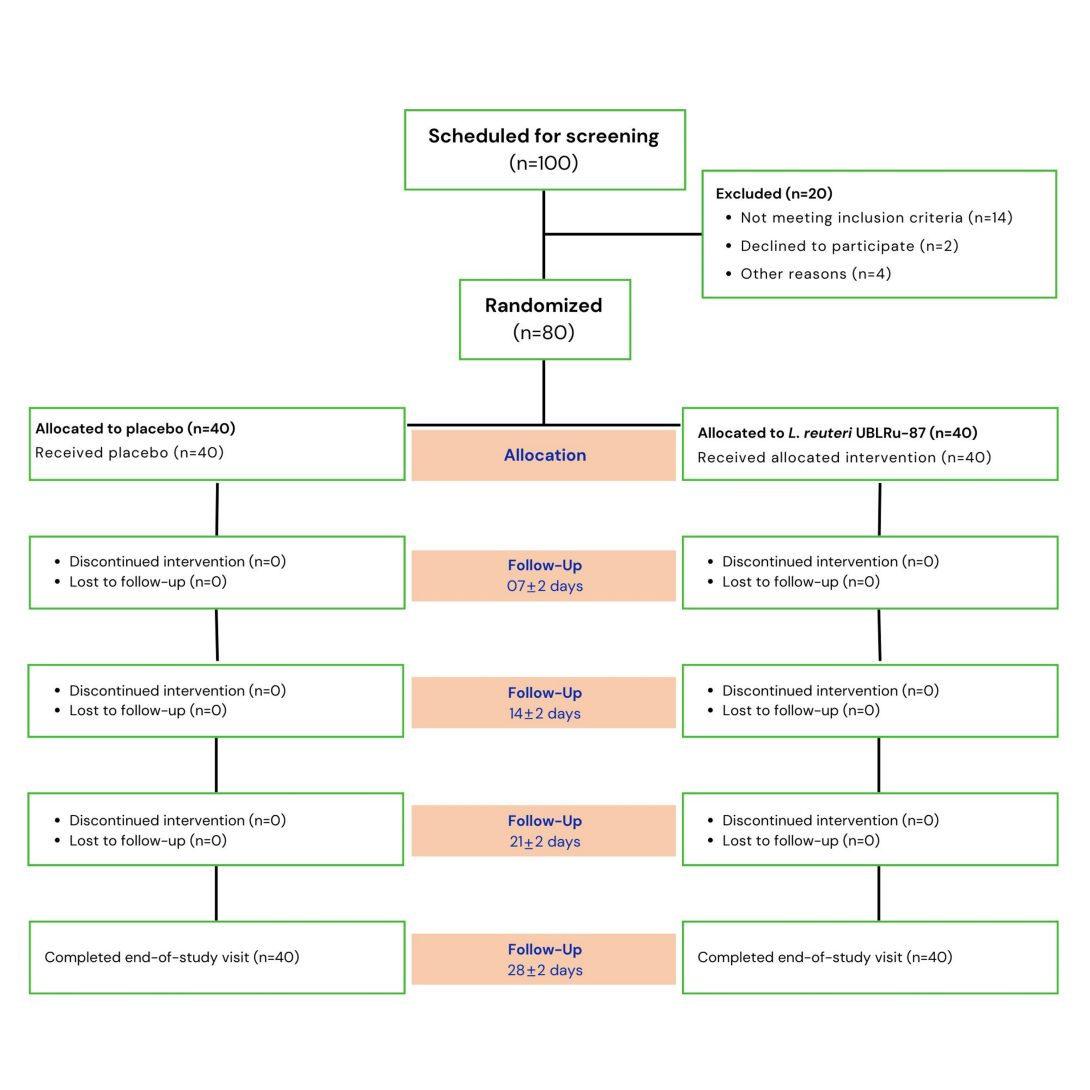
Schematic overview of the CONSORT flow diagram for the study

A total of 80 healthy infants diagnosed with colic pain were enrolled in the study after screening 100 infants. All the enrolled infants completed the study (Figure 1).* *The infants were divided into two groups, each consisting of 40 infants: the probiotic and the placebo groups. In the probiotic group, infants were administered five drops of* L. reuteri* UBL Ru-87 suspension containing 100 million CFU once daily. In the placebo group, infants were administered drops containing only the excipients, which were identical to the probiotic drops.

**Selection criteria

Inclusion Criteria

The study enrolled infants aged ≤4 months who met the criteria for infantile colic according to the Rome IV criteria, defined as experiencing crying episodes lasting three or more hours per day on at least three days per week within a seven-day period. Inclusion criteria included exclusively breastfed infants born full-term with a gestational age between 37 and 42 weeks, a birth weight of at least 2500 g, and an Apgar score greater than 8 at five minutes following birth. In addition, parents or caregivers had to give written informed consent, agree to share data, and adhere to scheduled follow-up visits as directed by the healthcare professional.

Exclusion Criteria

Infants were deemed ineligible for the study if they had any significant co-existing illness or major medical problem, had used antibiotics or acid suppressive therapy within the last 15 days, or had consumed probiotic supplements or probiotic-rich foods within the past two weeks that could potentially influence the study's endpoints.

Intervention and follow-up study

The treatment group received five drops of *L. reuteri* UBL Ru-87 probiotic suspension containing 100 million CFU once a day for 28 days (end of treatment (EOT), while the control group received five drops of identically looking placebo suspension. Assessments were performed at baseline and at the first, second, third, and fourth weeks of intervention. At study initiation, the parents/caregivers were given 28-day diaries to record colic symptoms on a daily basis. They were also instructed to use the visual analogue scale to record their quality of life (QOL) related to sleep quality and stress levels. A 10 cm VAS was used, with the scores ranging from 0 to 10. A score of 0 indicated “no effect or dissatisfaction," while a score of 10 indicated “very good effect or satisfaction” [17]. In addition, the parents were asked to note any adverse events, such as vomiting and constipation in the infants, and to contact the investigators if necessary.

Outcomes

The primary efficacy parameter was treatment success, defined as a decrease in the daily average crying time (minutes/day). Secondary parameters included identifying responders, defined as infants experiencing a 50% reduction in crying time from baseline, evaluating the reduction in other colic-associated symptoms such as fussiness, facial flushing, and burping, assessing the QOL for parents or caregivers, and analyzing fecal microbiota to investigate the impact of *L. reuteri* UBLRu-87 administration on gut microbiota composition.

Sample size

The sample size was determined considering an effect size (Cohen's d) of 0.5, a desired statistical power (1 - β) of 0.8, and a significance level (α) of 0.05 for a two-tailed test. The standard deviation (σ) of the outcome measure, which is the reduction in crying duration from Day 0 to Day 28, was estimated at 100 minutes. Given an anticipated detectable difference (Δ) of 50 minutes between the placebo and probiotic groups, the sample size per group was calculated using the formula for independent samples in RCT:

 n =((Zα/2​+Zβ​) ⋅ σ/∆​)2

Substituting the values of Zα/2 ≈1.96 minutes, Zβ​ ≈ 0.84, σ = 100 minutes, and Δ = 50 minutes yielded a required sample size of approximately 32 participants per group. To account for potential dropouts, the total sample size was adjusted to 80 participants, or 40 per group

Randomization

After obtaining signed, written informed consent from the parents/caretakers, infants meeting the inclusion/exclusion criteria were enrolled and randomly assigned to one of the two treatment arms using block randomization. Randomization numbers for the two treatment groups were generated using SAS 9.4 (Cary, NC, USA). The randomization process was performed by the principal investigator using opaque sealed envelopes, ensuring that the treatment assignments remained blinded to both the investigators and the parents/caregivers. Each envelope contained the assignment of the patient (probiotic or placebo treatment), with 40 envelopes for each group. Both treatment groups were similar in terms of age, sex, and weight of the patients, ensuring comparability.

Gut microbiota assessment

Faecal samples (10-15 g) were collected at baseline and day 28 (EOT) from infant diapers. DNA was extracted, and V3-V4 amplicons were generated using nested PCR. Library preparation involved end repair, dA-tailing, adapter ligation, and cleanup with SPRI beads, followed by limited cycle PCR for indexing. Libraries were quantified and sequenced on Illumina Miseq, producing 0.5M reads and 2 x 250 bp sequence reads per sample, generating FASTQ files. Raw sequence files from Illumina sequencing were imported into QIIME2 for demultiplexing and processing. Paired-end reads were trimmed and merged using Cutadapt and Vsearch tools. Representative sequences were used to generate feature tables and taxonomic classifications. Diversity analysis involved phylogenetic tree construction and core metrics calculation. Taxonomy abundance was visualized using Krona plots.

Statistical analysis

The study had no dropouts, ensuring consistency between intention-to-treat (ITT) and per-protocol (PP) populations. Data was analyzed using IBM SPSS Statistics for Windows, Version 26.0 (released 2019, IBM Corp., Armonk, NY). Numerical variables were expressed as mean ± standard deviation and categorical variables as frequency and percentage. Repeated-measure ANOVA assessed treatment effects on crying duration. Generalized estimating equation (GEE) analysed treatment and time impact on symptom reduction. Chi-square tests compared categorical variables (p < 0.05).

## Results

Demographic details 

The average age of the infants was 64.29 ± 19.59 days. Of the participants enrolled in the study, 51.25% (41) were female and 48.75% (39) were male. All the subjects included in the study were of Indian origin (Table 1). 

**Table 1:** Demographic data Values are presented as N (%) or mean ± SD.

Description			Placebo	Probiotic	p -value
Gender		Male	17 (42.5%)	22 (55%)	0.263
		Female	23 (57.5%)	18 (45%)	
Mode of delivery		Normal	31 (77.5%)	34 (85%)	0.390
		Caesarean	9 (22.5%)	6 (15%)	
Symptoms	Fussiness		40 (100%)	40 (100%)	
	Facial flushing		35 (87.5%)	36 (90%)	0.723
	Burping		35 (87.5%)	32 (80%)	0.363
	Frequent crying		40 (100%)	40 (100%)	
Weight on birth (kg)			3.12 ± 0.29	3.13 ± 0.27	0.852
Age (days)			64.15 ± 20.21	65.18 ± 19.92	0.820
Weight of infant on enrolment (kg)			4.63 ± 0.72	4.68 ± 0.81	0.754
Crying duration (minutes per day)			255.48 ± 38.26	258.2 ± 22.05	0.697
VAS score			1.2 ±0.41	1.27±0.45	0.437

Primary efficacy 

Crying Duration

**Figure 2:**
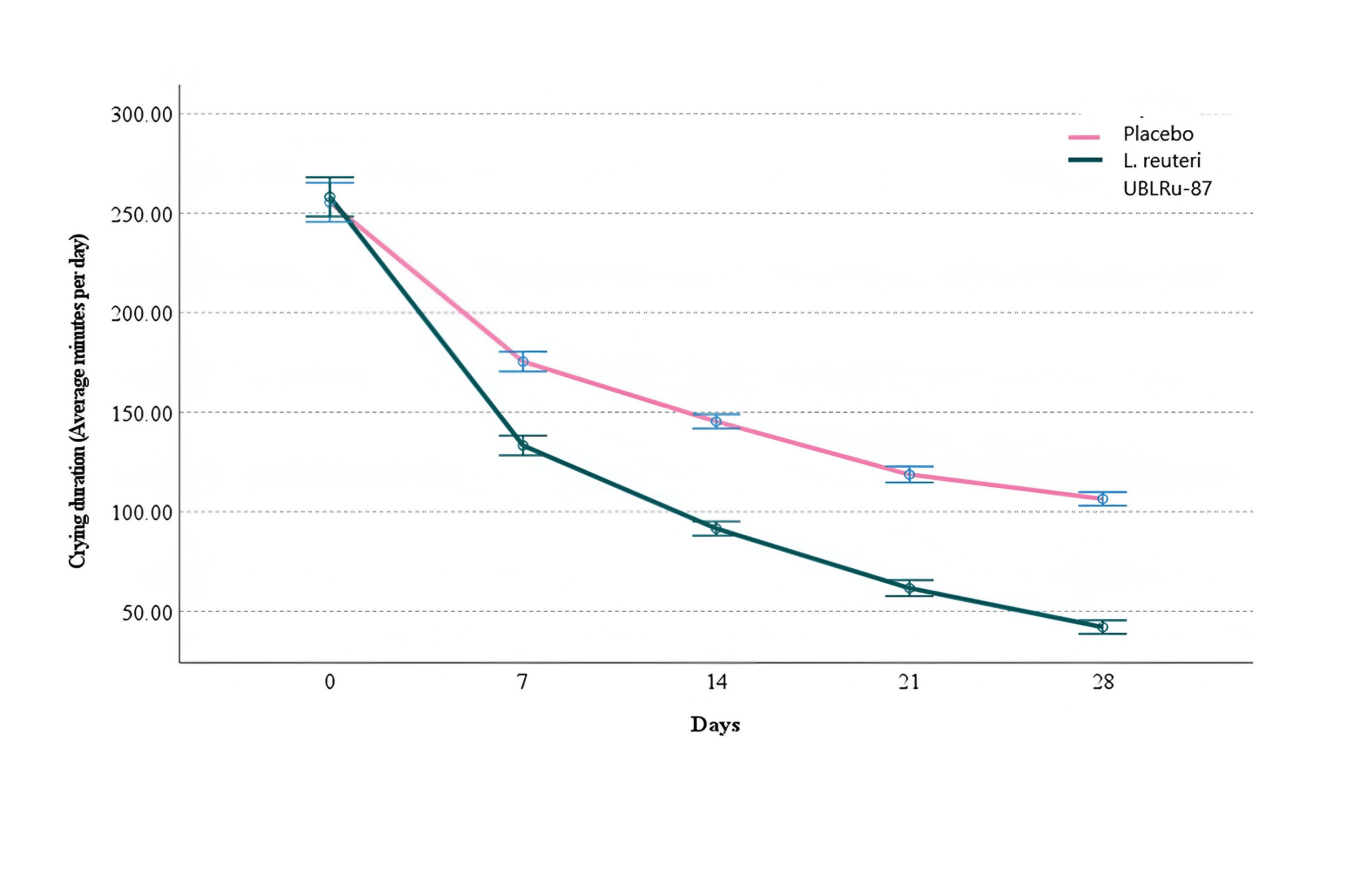
Crying duration on every visit

The treatment's success was based on the reduction in the duration of crying. In the* L. reuteri *UBLRu-87 treated group, the crying time decreased from 258.2 to 42.05 minutes/day by the end of treatment (EOT), whereas in the placebo group, it decreased from 255.48 to 106.50 minutes/day. The ANOVA test showed significant effects of both time (F (2.012, 156.955) = 136.183, p < 0.001, η² = 0.946) and treatment (F(2.012, 156.955) = 461.1, p < 0.001, η² = 0.857) on the crying duration, thereby demonstrating the efficacy of *L. reuteri* UBLRu-87 in bringing about a significant reduction in crying time as compared to placebo (Figure 2). 

Secondary efficacy

Responders

The number of responders, defined as those experiencing a 50% reduction in crying time from baseline, significantly increased in the *L. reuteri* UBLRu-87 group compared to the placebo group. On day 7, approximately 17 infants (42.5%) from the probiotic group and five infants (12.5%) from the placebo group were responders. By day 14, about 37 (92.5%) infants from the probiotic group and only nine infants (22.5%) from the placebo group had a 50% reduction in crying time. At the end of 28 days (EOT), all 40 infants (100%) from the probiotic group and 30 infants (75%) from the placebo group demonstrated a 50% reduced crying time. 

The probiotic group was at least five times more likely to achieve treatment success than the placebo group, with the odds ratio of 5.174 (95% CI 1.676-15.975) and 42.481 (95% CI 10.57-170.738) by the end of the first and second weeks, respectively. These results indicate significantly greater odds of treatment success (50% reduction) in the probiotic group compared to the placebo group on day 7 and day 14. However, by day 21 and day 28, all participants had achieved the 50% reduction threshold, making the GEE test inappropriate for these time points.

Other symptoms associated with colic pain

At enrolment, all of the study infants experienced fussiness and frequent crying symptoms. In the placebo group, approximately 87.5% experienced facial flushing and 80% had burping, whereas 90% in the probiotic group had facial flushing and 87.5% had burping. A significant reduction in symptoms was observed in the probiotic-treated groups starting from the first week of intervention, except for fussiness. At the end of the study, the probiotic-treated group demonstrated a statistically significant reduction in all symptoms compared to the placebo-treated group (p < 0.05) (Table 2).

**Table 2:** Comparison of placebo and L. reuteri UBLRu-87 on other colic symptoms Values are presented as N (%) or mean ± SD; p-values < 0.05 = *, and p-values < 0.001 = **.

Symptoms		Samples (N = 80)		p-value
		Placebo (n = 40)	Probiotic (n = 40)	
Fussiness	Day 0	40 (100%)	40 (100%)	
	Day 7	37 (92.5%)	32 (80%)	0.105^*^
	Day 14	32(80%)	15(37.5%) **	<0.001^*^
	Day 21	26 (65%)	9 (22.5%) **	<0.001^*^
	Day 28	18(45%)	2 (5%) **	<0.001^#^
Frequent crying	Day 0	40 (100%)	40 (100%)	
	Day 7	31 (77.5%)	19 (47.5%) **	<0.001 **
	Day 14	25 (62.5%)	14 (35%) *	0.014 *
	Day 21	17 (42.5%)	8 (20%) *	0.030 *
	Day 28	11 (27.5%)	4 (10%) *	0.042 *
Facial flushing	Day 0	36 (90%)	35 (87.5%)	0.723
	Day 7	35 (87.5%)	24 (60%) *	0.005 *
	Day 14	24 (60%)	8 (20%) **	<0.001 **
	Day 21	16 (40%)	1 (2.5%) **	<0.001 **
	Day 28	11 (27.5%)	0 **	<0.001 **
Burping	Day 0	35 (87.5%)	32 (80%)	0.363
	Day 7	33 (82.5%)	19 (47.5%) **	<0.001 **
	Day 14	19 (47.5%)	1 (2.5%) **	<0.001 **
	Day 21	14 (35%)	0 **	<0.001 **
	Day 28	7 (17.5%)	0 *	0.006 *

Quality of life

**Figure 3:**
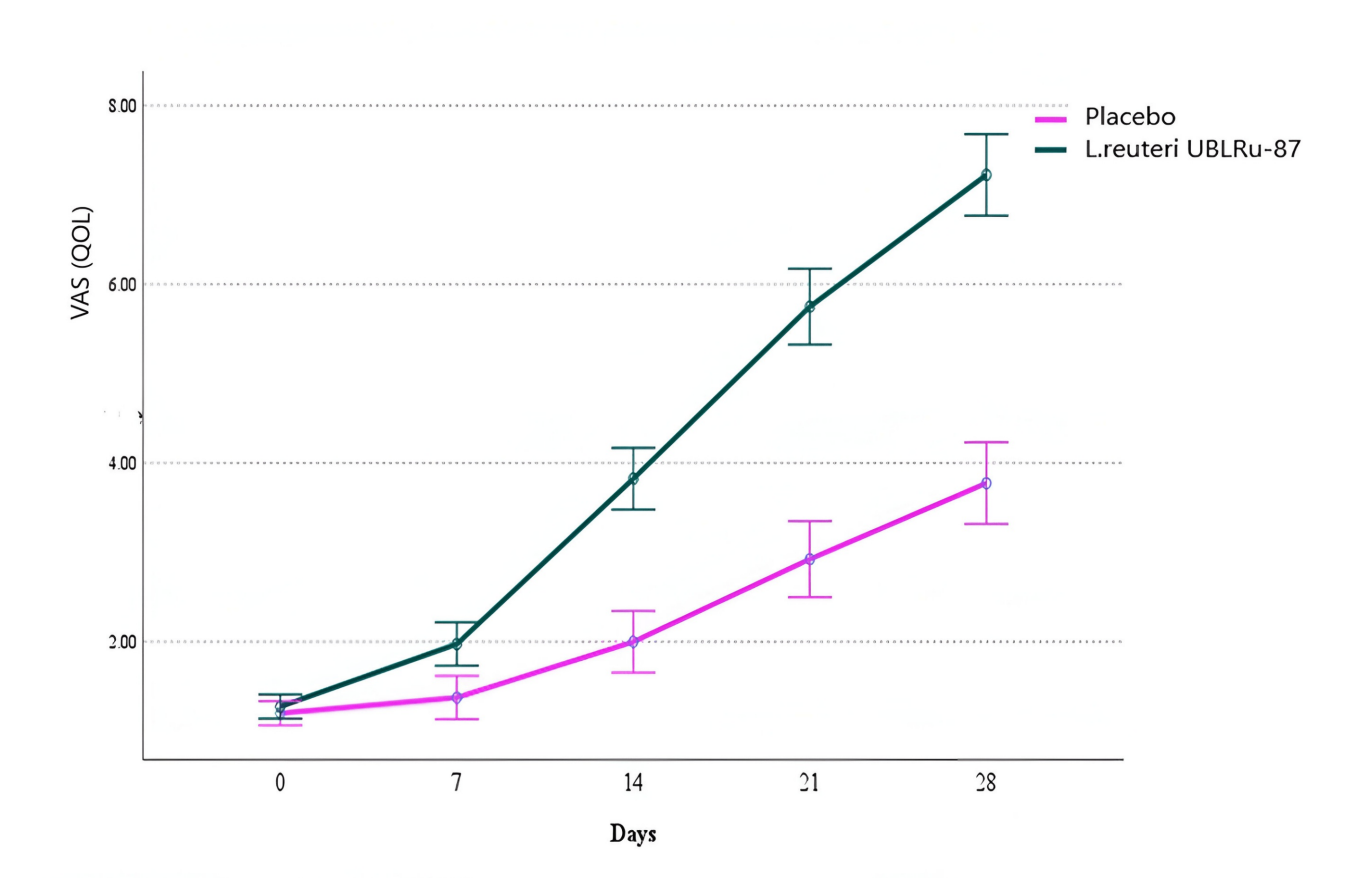
Quality of life determined with the visual analog scale (VAS) score

The QOL for parents/caretakers improved significantly in the* L. reuteri *UBLRu-87 group (p < 0.001, η² = 0.523). VAS scores in the probiotic group increased from 1.27 ± 0.45 at baseline to 7.23 ± 1.29 by week four, compared to 1.2 ± 0.41 to 3.78 ± 1.59 in the placebo group (Figure 3).

Gut microbiota composition

**Figure 4:**
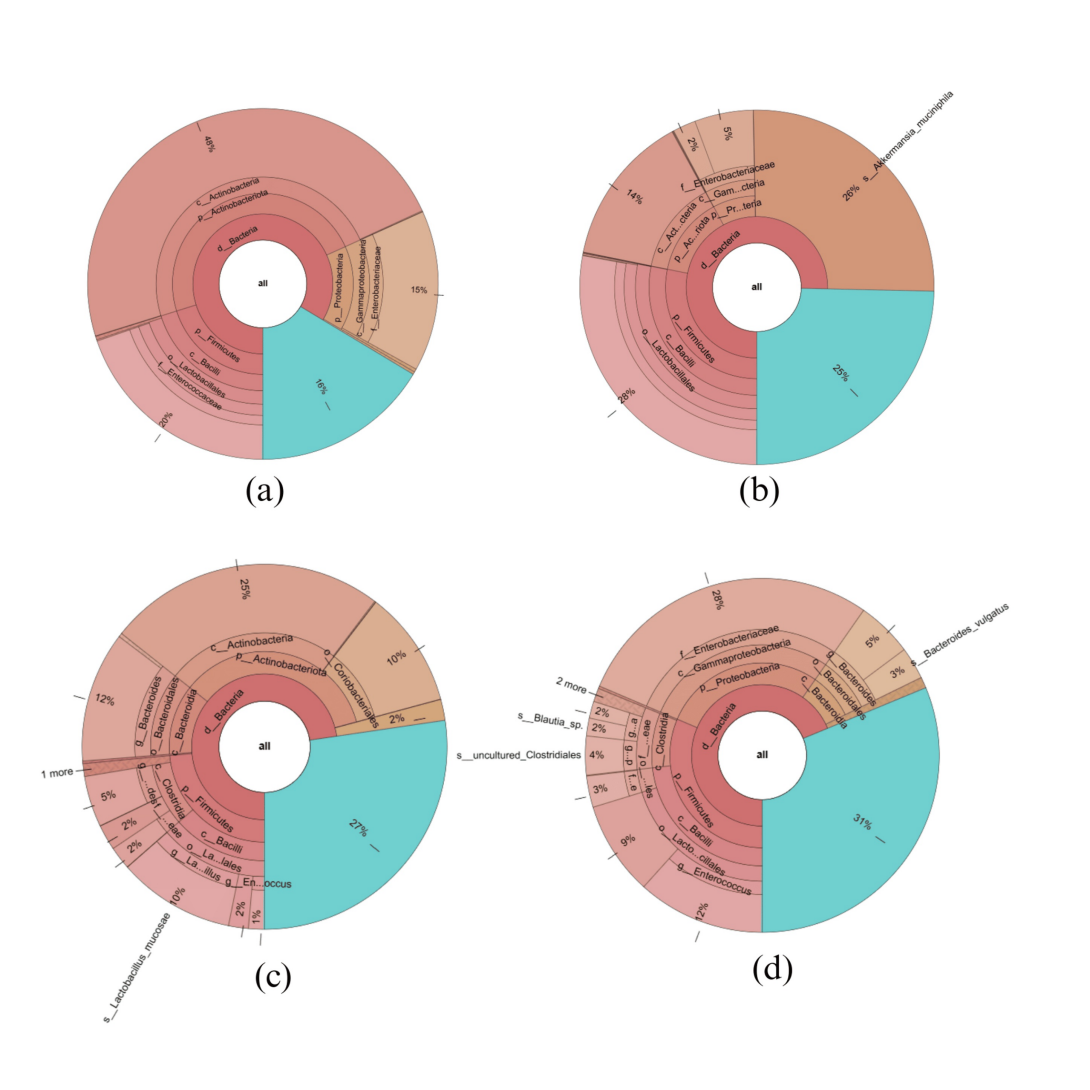
Krona graph representing the taxonomic assignment of microbiota in (a) baseline placebo, (b) EOT placebo, (c) baseline probiotic, and (d) EOT with L. reuteri UBLRu-87 groups

The analysis of fecal samples revealed variation in gut microbiota profiles. Both groups predominantly had *Actinobacteriota, Firmicutes*, and *Proteobacteria *spp., with the probiotic group additionally having Bacteroidetes at baseline. The placebo group had* Staphylococcus, Bifidobacterium,* and *E. coli-Shigella* spp. while the probiotic group had *Bifidobacterium*, *Escherichia-Shigella*, *Enterococcus*, and *Bacteroides*. After 28 days, the placebo group's microbial pattern remained the same, except for the presence of Akkermansia in 30% of the samples. The probiotic group had a higher Firmicutes/Bacteroidetes ratio, indicating beneficial bacterial growth. Pathogenic genera such as *Escherichia-Shigella*, *Rubrobacter*, *Rothia*, *Staphylococcus*, *Collinsella*, *Eubacteria*, and *Clostridiodes *decreased with a predominance of other bacterial genera like *Enterococcus* (22%),* Streptococcus *(11%), *Clostridium* (2%), uncultured *Clostridiotes*(6%), *Ruminococcus* (1%), *Blautia *(3%), and *Veillonella *(3%). A Krona graph was used to illustrate the genus-level taxonomic classification (Figure 4). Alpha diversity was assessed to determine the richness and abundance of microbial species within the group, measured by the Shannon index. Statistically significant difference in richness and abundance was observed in the probiotic group compared to the placebo group. The Shannon index value was found to be higher in the probiotic group after treatment compared to baseline, indicating increased species richness (data not shown). A higher H-index value signified greater species diversity, corroborating the taxonomic variations observed in the probiotic group after treatment with* L. reuteri* UBLRu-87.

Adverse events (AEs)

No adverse events were observed in either of the treatment groups, indicating that the probiotic and the placebo were well tolerated.

## Discussion

In this double-blind, placebo-controlled study, *L. reuteri* UBL Ru-87 supplementation significantly reduced crying time and other symptoms, such as burping and fussiness, in colicky breastfed infants. The efficacy of our findings is consistent with a few earlier studies that have examined the effects of* L. reuteri* DSM 17938 on managing infantile colic [16-21]. In our study, statistical significance between probiotic and placebo groups was achieved as early as seven days after initiating probiotic therapy compared to the placebo. A similar trend was observed when analyzing the number of responders to the treatment. This superior efficacy aligns with the findings of Chau et al. (2015) and Szajewska et al. (2013), who reported similar effects with the strain *L. reuteri* DSM 17938 [3,18]. In our study, the breastfeeding mothers were not on a cow protein elimination diet, similar to trials conducted by Szajewska et al. (2013) and Chau et al. (2015), further corroborating that hypersensitivity to cow’s milk protein is not a leading cause of colic pain. Along with the improvement in colic symptoms, there was also an observed enhancement in the QOL of parents, consistent with the results of other researchers [3,18,22]. It is reported that infants with colic pain have high levels of *Acinetobacter, Clostridium, *and *Escherichia* spp. [23] and low levels of *Bifidobacterium* [8]. *Escherichia* is known for its gas-producing properties and its production of inflammatory lipopolysaccharides (LPS), which lead to increased gut permeability, resulting in hepatic and systemic inflammation that may manifest as colic pain in infants. Bacteria belonging to the Bacteroidetes and Firmicutes phyla, including butyrate-producing bacteria, were found to be reduced in colicky infants [24]. Butyrate is known for its anti-inflammatory properties, its role in fueling enterocytes, and its pain-reducing effects in the adult intestine [25].

In our study, treatment with *L. reuteri *UBLRu-87 led to increased gut microbiota from the *Lachnospiraceae (Blautia, Ruminococcus) *and *Veillonellaceae (Veillonella) *families. These species have anti-inflammatory properties and produce short-chain fatty acids (SCFAs) and antimicrobial peptides [26-30]. In addition, higher levels of other beneficial bacteria *(Bifidobacteria, Streptococci, *and* Firmicutes)* and reduced harmful bacteria (*Enterococci *and* Staphylococci*) were observed. The observed positive shifts in the fecal microbiome further support the role of *L. reuteri *UBLRu-87 in alleviating symptoms. 

However, the study presents a few limitations in that it was restricted to breastfed infants diagnosed with colic. Moreover, maternal dietary factors like soy, dairy, or caffeine, which can contribute to colic pain in infants, were not taken into consideration. 

In summary, administering Lactobacillus reuteri UBLRu-87 to breastfed infants with colic resulted in a notable reduction in colic pain and related symptoms, offering a promising, well-tolerated approach to improving infant comfort and parental QOL.

## Conclusions

Our findings provide compelling evidence for the beneficial effects of* L. reuteri *UBLRu-87 in managing infantile colic. The observed reductions in crying time, fussiness, and burping, alongside improvements in parental QOL, emphasize the potential benefits of *L. reuteri* UBLRu-87 supplementation. Furthermore, the positive shifts in fecal microbiome composition, such as the increase in beneficial bacteria like Bifidobacteria and Firmicutes, and the reduction in harmful bacteria like *Enterococci*, *Streptococci*, and *Staphylococci* reinforce the therapeutic properties of *L. reuteri *UBLRu-87. 
